# Identifying core genes in keloid and investigating immune infiltration and pan-cancer associations using eQTL and machine learning

**DOI:** 10.1093/bioadv/vbaf076

**Published:** 2025-04-26

**Authors:** Xiaoyuan He, Yang Song

**Affiliations:** Second Clinical College, Changzhi Medical College, Changzhi, Shanxi 046000, China; Laboratory Animal Center, Changzhi Medical College, Changzhi, Shanxi, 046000, China

## Abstract

**Motivation:**

Keloid is a fibroproliferative skin disorder characterized by excessive fibroblast proliferation and abnormal extracellular matrix accumulation. It manifests as continuous growth, redness, itching, and pain, with a high recurrence rate. The pathogenesis of keloid is complex, with genetics and gene mutations increasingly recognized as critical risk factors. This condition exhibits familial predisposition and clustering, with individuals of darker skin tones at greater risk. To elucidate the genetic factors underlying keloid development, this study integrates bioinformatics and Mendelian randomization (MR) approaches to identify core genes associated with keloid, providing novel insights into its pathogenesis, treatment, and prognosis.

**Results:**

Bioinformatics and Mendelian randomization analyses identified two intersecting genes, CCND2 and KLF4, as core genes associated with keloid. MR analysis revealed that CCND2 is causally associated with keloid [inverse variance weighted (IVW) odds ratio (OR): 1.410; 95% confidence interval (CI): 1.001–1.985, *P* = .049], indicating it is a risk factor, while KLF4 is inversely associated with keloid (IVW OR: 0.492; 95% CI: 0.290–0.835, *P* = .009). Both intersecting genes exhibit a causal relationship with keloid, identifying them as two core genes. Specifically, CCND2 is recognized as a risk factor for keloid, while KLF4 functions as a protective factor against keloid formation. Validation analyses were conducted on these two core genes, revealing significant differences in KLF4 expression within the validation cohort.

**Availability and implementation:**

Firstly, bioinformatics analysis identified differentially expressed genes (DEGs) from the keloid GEO datasets. Secondly, MR was applied to eQTL and keloid GWAS datasets to identify candidate genes. Overlapping genes were derived by intersecting DEGs with MR candidate genes. Causal relationships between overlapping genes and keloids were analyzed using five MR methods, identifying core genes significantly associated with keloid pathogenesis. Cochran’s Q test and MR-Egger intercept analysis evaluated heterogeneity and pleiotropy in MR results. GO, KEGG, and GSEA enrichment analyses were conducted to explore core gene functions. Finally, validation and TCGA pan-cancer analyses were conducted on the core genes.

## 1 Introduction

Keloids are benign cutaneous tumors resulting from fibroproliferative disorders following skin injury. They are characterized by the abnormal proliferation of fibroblasts and excessive extracellular matrix (ECM) deposition (Li *et al.* 2024). Keloids exhibit persistent, invasive, tumor-like growth patterns and can occur on any part of the body, though most commonly found on the anterior chest. These lesions typically arise from abnormal wound healing triggered by skin injury or inflammation, leading to uncontrolled fibroblast proliferation and ECM accumulation resistant to spontaneous regression (Lee and Jang 2018). The incidence of keloids varies by race, with individuals of darker skin tones demonstrating a significantly higher predisposition than those with lighter skin tones (LeFlore 1980). Africans and individuals of African descent have the highest prevalence, whereas the rate among Caucasians is relatively low, reaching only 0.09% in the United Kingdom (Bloom 1956). This racial disparity underscores the potential genetic components contributing to keloid formation. Keloids significantly impact patients’ quality of life and mental health due to functional impairments, including contractures, neurogenic itching and pain, cosmetic disfigurement, and a high recurrence rate after surgical excision (Chiang *et al.* 2016, Delaleu *et al.* 2023). Genetics plays a critical role in keloid development, manifesting through racial disparities and familial clustering. Individuals with familial keloids are more prevalent than those with sporadic cases, although the specific genetic variants responsible remain largely undefined (Glass 2017). Understanding the genetic mechanisms underlying keloid formation is essential for developing effective therapeutic strategies and improving patient outcomes. This study integrates bioinformatics and Mendelian randomization (MR) methods to identify core genes associated with keloid pathogenesis. Bioinformatics is an interdisciplinary field that combines information science and statistical methodologies to analyze and interpret large-scale biological data using specialized software and online platforms (Uesaka *et al.* 2022). Bioinformatic approaches have been widely used to identify key genes and pathways involved in various diseases, such as psoriasis, atopic dermatitis, melanoma, and gliomas (Wu *et al.* 2022, Zuo *et al.* 2022, Lin *et al.* 2023, Sheng *et al.* 2023). MR is a statistical method that uses genetic variants as instrumental variables to infer causal relationships between exposure factors and outcomes (Sekula *et al.* 2016, Birney 2022). The core principle of MR leverages the random allocation of genetic variations as instrumental variables to investigate the causal links between exposures and diseases. This study utilized single nucleotide polymorphisms (SNPs) as instrumental variables. SNPs are DNA sequence variations caused by single-nucleotide alterations at the genomic level (Steventon-Jones *et al.* 2022). These variations represent humans’ most common form of genetic variation and are critical for understanding genetic diversity, disease susceptibility, drug responses, and other biological processes. Expression Quantitative Trait Loci (eQTLs) are genetic loci that influence gene expression levels, with most being SNPs. eQTL studies establish associations between genotypes and gene expression levels in individual samples, enabling the identification of gene expression variations linked to diseases and providing valuable insights into the mechanisms underlying disease onset and progression (Li *et al.* 2012b, Zhu *et al.* 2016). eQTL databases store and analyze these associations, facilitating research into the genetic regulation of gene expression and its implications for disease. In the MR analysis conducted in this study, the eQTL database served as the exposure factor, SNPs were used as instrumental variables, and keloid genome-wide association study (GWAS) data were used as the outcome. This approach was utilized to infer causal relationships between gene expression and keloid disease.

Herein, we used bioinformatic methods to identify differentially expressed genes (DEGs) associated with keloids. Next, we applied MR to analyze eQTL data and keloid GWAS datasets to identify candidate genes. We obtained a set of overlapping genes by intersecting the DEGs and MR-identified genes. Further MR analysis of these overlapping genes confirmed their causal relationship with keloid, designating them as core genes. To ensure the robustness of our findings, we conducted several validation steps, including heterogeneity analysis using Cochran’s *Q* test, pleiotropy analysis using the MR-Egger intercept method, and leave-one-out sensitivity analysis. These validations aimed to identify potential biases and evaluate the sensitivity of our results. Following the MR analysis, functional and pathway enrichment analyses were performed on the core genes to explore their biological roles in keloid development. Additionally, immune cell infiltration analysis was conducted to investigate the immunological mechanisms involving the core genes. Finally, we performed validation and pan-cancer analyses using The Cancer Genome Atlas (TCGA) to corroborate the relevance of these core genes further and assess their potential implications in a broader cancer context. These comprehensive analyses provide a robust foundation for future research to develop targeted therapies and improve patient outcomes in keloid management.

## 2 Methods

### 2.1 GEO data download

We searched the Gene Expression Omnibus (GEO) database (https://www.ncbi.nlm.nih.gov/geo/) by using the keyword “keloid” and selected the GSE92566, GSE121618, GSE7890, and GSE145725 datasets for this study. The datasets were downloaded, organized, and preprocessed as follows. GSE92566 (GPL570) includes four disease samples and three control samples. GSE121618 (GPL21185) includes five disease samples and six control samples. GSE7890 (GPL570) includes five disease samples and five control samples (all included samples are pretreatment samples with hydrocortisone). GSE145725 (GPL16043) includes nine disease samples and 10 control samples. The datasets underwent correction and normalization using Principal Component Analysis (PCA), implemented with the “limma” and “sva” packages in R. Use the “avereps” and “normalizeBetweenArrays” function to correct the data and normalize the expression data between different samples.

To facilitate further analysis, the datasets were grouped as follows: The GSE92566 and GSE121618 datasets were merged and designated as the training cohort. The GSE7890 and GSE145725 datasets were merged and designated as the validation cohort. The training and validation cohorts were derived from two different platform files. Cross-platform dataset analysis encompasses a broader range of experimental conditions and sample types, enhancing the ability to validate the universality of results. Integrating datasets from different platforms increases the amount of research data, thereby improving the reliability of statistical analyses and reducing the impact of random errors. Additionally, diverse sample sources were used to reflect biological variation better and provide more comprehensive biological coverage. To ensure the robustness of the statistical results, the dataset with the larger sample size was designated as the validation cohort.

### 2.2 Identification of differentially expressed genes (DEGs)

The data were first organized according to the grouping information of the samples, and the treatment and control group data were processed accordingly. The expression level of each gene was calculated across samples, and differences between the treatment and control groups were compared. The differential expression level and statistical significance of each gene were estimated, and the DEGs were identified based on these criteria. The false discovery rate (FDR) correction method was applied to adjust the *P*-values. Genes with a corrected *P*-value < .05 and an absolute value of log2 fold change (|logFC|) > 0.585 were classified as keloid DEGs. In this process, the “eBayes” function was primarily utilized for differential expression analysis. In differential expression analysis, a |logFC| threshold of 0.585 is often chosen to balance the identification of a larger number of DEGs with maintaining statistical significance. This threshold is informed by prior experience and the distribution of the data, enabling the detection of genes with relatively small but biologically meaningful expression changes between groups. After identifying the DEGs, their expression levels were extracted, and visualizations, including a heatmap and a volcano plot, were generated to represent the results.

### 2.3 Gene ontology (GO) and Kyoto Encyclopedia of Genes and Genomes (KEGG) enrichment analysis of DEGs

GO and KEGG enrichment analyses were performed on the identified DEGs to explore their functional and pathway implications in keloid biology. Results were visualized using GO and KEGG bubble charts.

### 2.4 Acquisition of eQTL data and keloid GWAS data

eQTL data were obtained from the GWAS Catalog (https://gwas.mrcieu.ac.uk/). SNPs showing strong associations with the exposure data (*P*-value threshold of 5 × 10^−8^) were selected as instrumental variables. Linked SNPs in linkage disequilibrium were excluded, and an F-test was applied to filter out weak instrumental variables using an F-value threshold > 10. This process generated high-confidence exposure data for eQTL analysis. Keloid-related GWAS data were retrieved from the same website using the keyword “Keloid.” The identified GWAS dataset, designated with the GWAS ID ebi-a-GCST90018874, comprised 24 197 210 SNPs, 668 disease samples, and 481 244 control samples.

### 2.5 Mendelian randomization analysis of gene eQTL and keloid

MR analysis was performed using processed eQTL data as the exposure dataset and keloid GWAS data (GWAS ID: ebi-a-GCST90018874) as the outcome dataset. The analysis was conducted in the R programming language using the “TwoSampleMR” package. The results of the MR analysis identified candidate genes potentially associated with keloid, enabling an investigation of causal relationships between genetic variations influencing gene expression and the risk of developing keloid.

### 2.6 Identification of overlapping genes

The DEGs identified from the bioinformatics analysis were intersected with the candidate genes obtained from the MR analysis. The overlapping genes resulting from this intersection were identified, and Venn diagrams were created to represent the overlap between these two gene sets visually.

### 2.7 Mendelian randomization analysis of overlapping genes and keloid

MR analysis was performed on the overlapping genes using their eQTL data as the exposure dataset and the keloid GWAS data (GWAS ID: ebi-a-GCST90018874) as the outcome dataset. Five MR methods were used to ensure robust results: MR Egger, Weighted Median, inverse variance weighted (IVW), Simple mode, and Weighted mode. MR Egger detects and adjusts for horizontal pleiotropy by introducing an intercept term to estimate the direction and magnitude of pleiotropic bias. Weighted Median provides reliable results in the presence of outliers and extreme values. IVW averages the estimates of instrumental variables weighted by their variances, offering a comprehensive and reliable causal effect estimate. Simple Mode computes an average of all instrumental variables without additional weighting or adjustment. Weighted Mode assigns different weights to instrumental variables based on their strength of association with the exposure variable. Among these methods, IVW is the most widely used in MR analyses due to its ability to provide stable and reliable causal effect estimates in most cases. The results of the MR analysis were assessed using *P*-values and odds ratios (ORs) and further evaluated through heterogeneity analysis, pleiotropy tests, and visualizations such as scatter plots, forest plots, and leave-one-out sensitivity analysis. Genes with a *P*-value < .05 from the IVW method and consistent OR directions across all five methods were identified as the core genes associated with keloid.

### 2.8 Functional and pathway enrichment analyses of core genes

Functional and pathway enrichment analyses were performed on the core genes to explore their biological roles in keloid development.

### 2.9 Immune cell infiltration analysis

Immune cell infiltration analysis was performed using the CIBERSORT method, a deconvolution algorithm based on support vector regression. This method estimates the proportions of various cell types within complex tissue samples. The analysis used the “e1071” and “limma” packages in R software. Immune infiltration analysis was performed on the combined training cohort data to explore the immune landscape of keloid. This analysis quantified the presence and abundance of various immune cell types within keloid samples. The results were visualized using a bar chart to illustrate the distribution of immune cell infiltration. Additionally, a correlation analysis was conducted between the core genes associated with keloid and the immune cell types identified in the infiltration analysis. The correlations were visualized to identify potential associations between specific immune cells and the expression levels of the core genes. This investigation aimed to elucidate the immune-mediated mechanisms underlying keloid development and progression, potentially revealing novel therapeutic targets or biomarkers for disease management.

### 2.10 Validation cohort differential analysis

The previously merged and normalized GSE7890 and GSE145725 datasets were used as the keloid validation cohort. Gene expression data files were processed to extract the expression levels of each gene in individual samples. Specifically, the expression levels of core genes were examined to determine whether they exhibited significant differences within the validation cohort. The Wilcoxon rank-sum test was performed to compare gene expression levels between different sample types, and the significance levels were annotated on the resulting visualizations.

### 2.11 TCGA pan-cancer and prognostic analysis of core genes

To assess the potential involvement of core genes in tumorigenesis, progression, and prognosis, their expression levels were analyzed across datasets from The Cancer Genome Atlas (TCGA). This large-scale, multi-omics cancer genome project provides extensive public data resources. Data and clinical information related to core genes and 33 cancer types were downloaded from the TCGA database (https://portal.gdc.cancer.gov). The expression levels of transcripts corresponding to core genes were measured in specific samples. Statistical analysis was conducted using R software, and results with a *P*-value < .05 were considered statistically significant.

## 3 Results

### 3.1 Differentially expressed gene analysis

**Figure 1.**
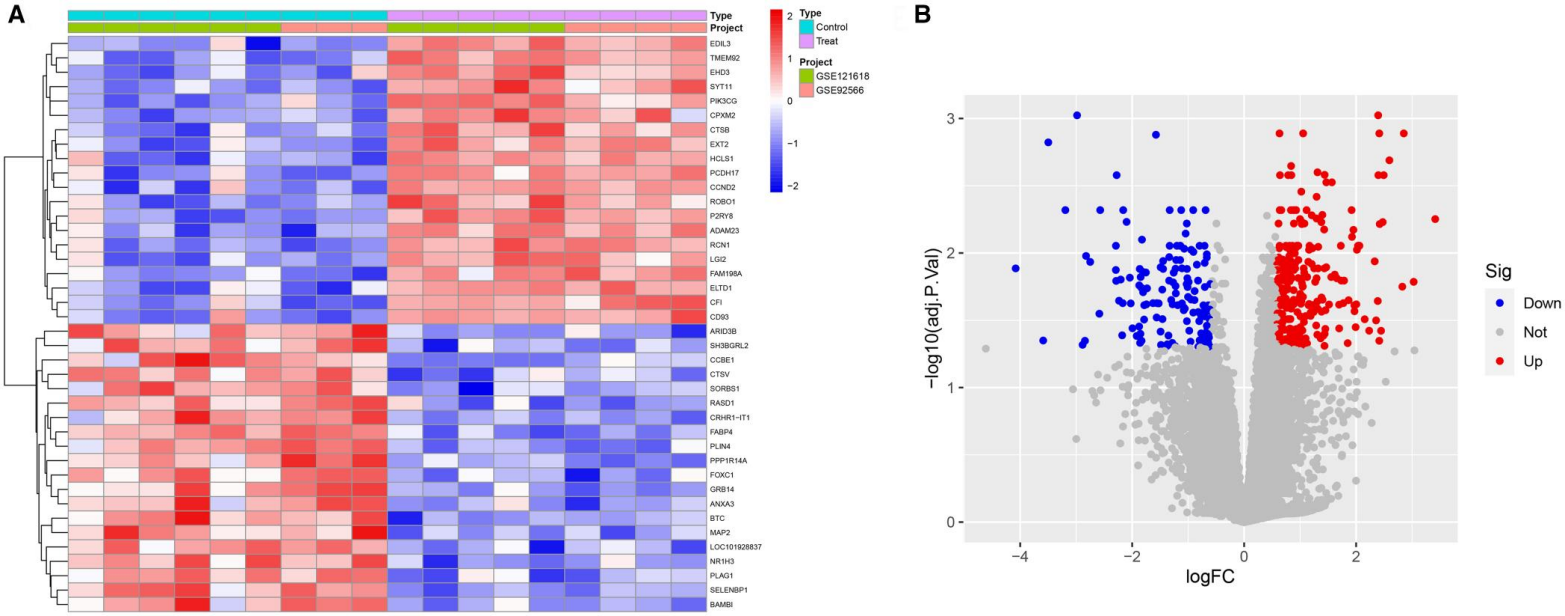
(A) Heatmap of DEGs. (B) Volcano plot of DEGs.

Differential gene expression analysis of the training cohort identified 244 upregulated and 137 downregulated genes in keloid samples compared to controls, reflecting substantial transcriptional alterations associated with keloid development. To visualize these results, a heatmap (highlighting the top 20 most significantly upregulated and downregulated genes) and a volcano plot were generated (Fig. 1A and B).

### 3.2 GO and KEGG enrichment analysis of DEGs

**Figure 2.**
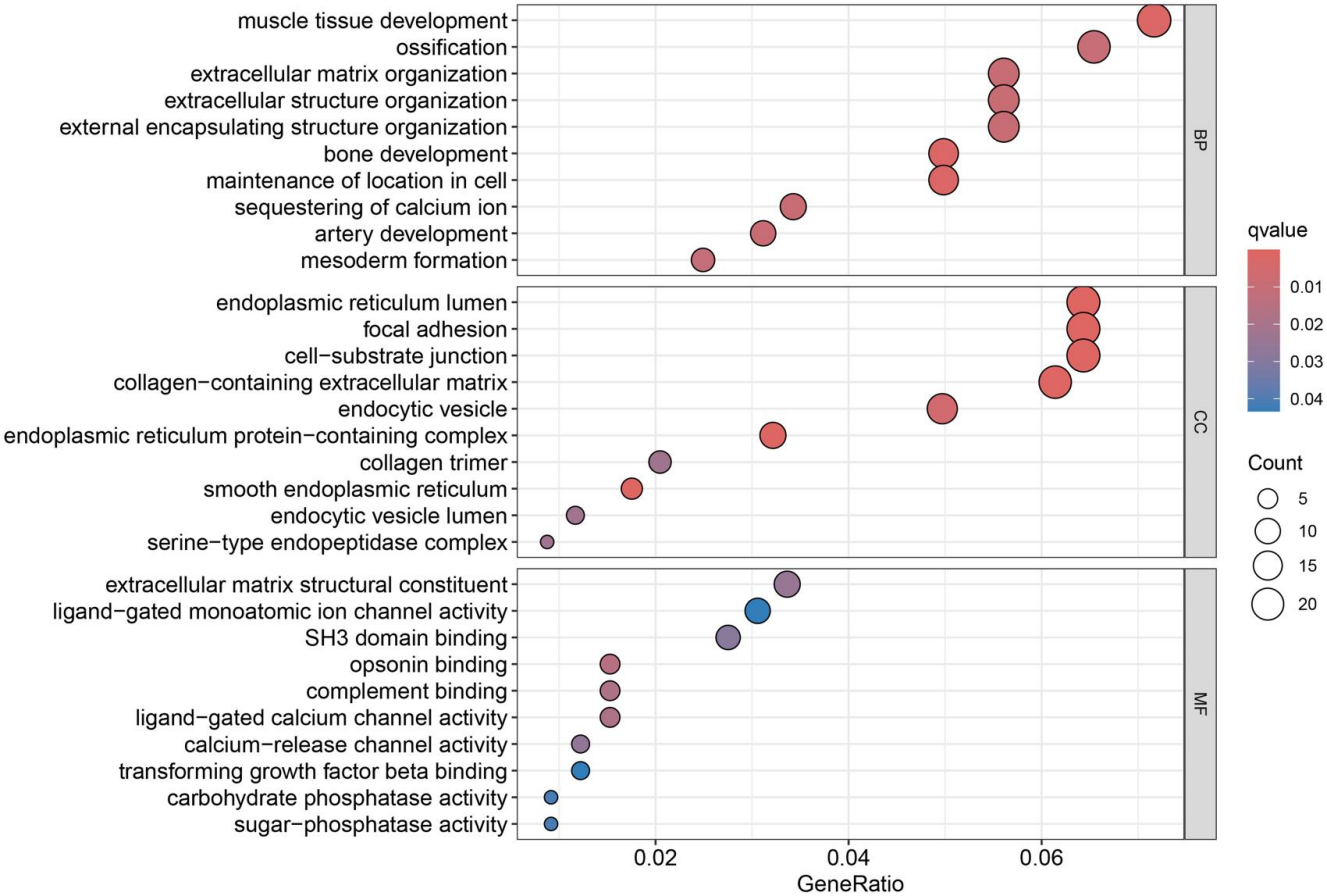
GO enrichment analysis of DEGs. The vertical axis represents pathway names, the horizontal axis represents the proportion of genes, the size of the circles represents the number of DEGs enriched in each pathway, and the color of the circles represents the significance of enrichment.

**Figure 3.**
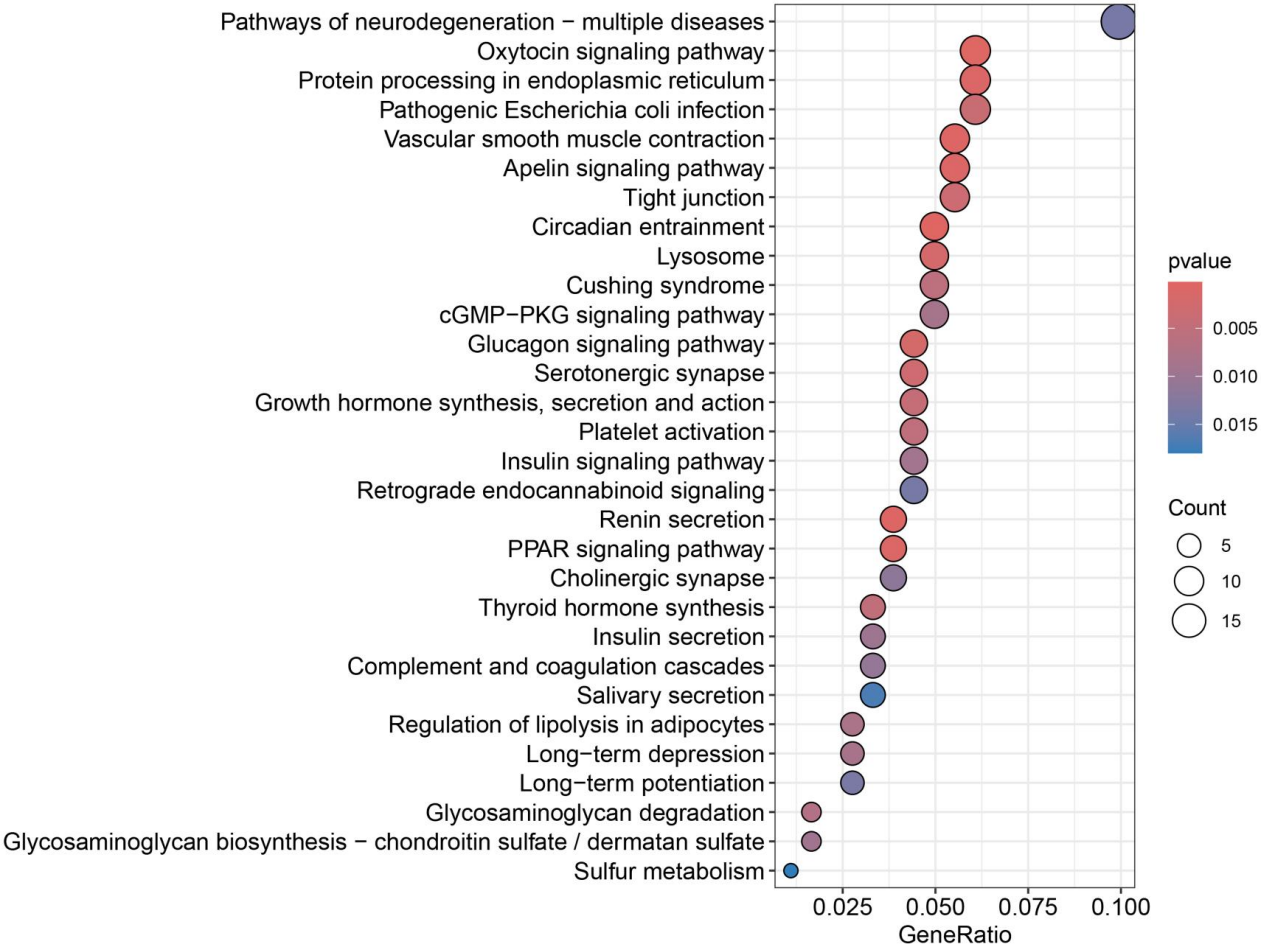
KEGG enrichment analysis of DEGs.

GO enrichment analysis on DEGs (Figs 2 and 3) revealed that in biological process (BP), DEGs are significantly enriched in pathways such as muscle tissue development, ossification, extracellular matrix organization, extracellular structure organization, and bone development. In the cellular component (CC), DEGs are significantly enriched in the endoplasmic reticulum lumen, focal adhesion, and cell-substrate junction. In molecular function (MF), DEGs are significantly enriched in extracellular matrix structural constituent. KEGG enrichment analysis on the DEGs revealed significant enrichment in pathways such as Circadian Entrainment, Oxytocin Signaling Pathway, Vascular Smooth Muscle Contraction, Renin Secretion, Apelin Signaling Pathway, and Protein Processing in the Endoplasmic Reticulum.

### 3.3 Analysis of gene eQTL and keloid MR

MR analysis was performed using the processed gene eQTL exposure data and keloid GWAS outcome data, identifying 93 high-risk candidate genes and 109 low-risk candidate genes.

### 3.4 Acquisition of keloid intersection genes

**Figure 4.**
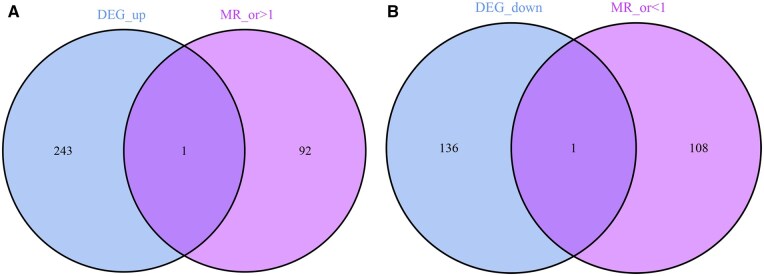
Venn diagrams. (A) Venn diagram showing overlapping upregulated DEGs and high-risk eQTL MR candidate genes. (B) Venn diagram showing overlapping downregulated DEGs and low-risk eQTL MR candidate genes.

The intersection of DEGs and MR-identified candidate genes yielded two intersection genes. Specifically, the intersection of upregulated DEGs with high-risk candidate genes identified CCND2. The intersection of downregulated DEGs with low-risk candidate genes identified KLF4. The Venn diagrams illustrating these overlaps are shown in Fig. 4A and B.

### 3.5 Intersection genes and keloid MR analysis

#### 3.5.1 Analysis of Mendelian randomization results

Regarding keloid development, MR analysis was conducted for the two intersection genes, CCND2 and KLF4. Five MR methods were used, resulting in *P*-values, beta coefficients, and ORs, as summarized in Table 1. Among these methods, the IVW method was the most significant. A summary of the MR results is presented in Table 2. Both intersecting genes exhibited *P*-values below .05, indicating a significant causal relationship between each gene and keloid. Consequently, CCND2 and KLF4 were identified as core genes. For CCND2, the beta coefficient was positive, and the OR exceeded 1, suggesting that higher expression levels of CCND2 are associated with an increased risk of keloid formation. Conversely, for KLF4, the beta coefficient was negative, and the OR was <1, indicating that increased expression of KLF4 reduces the risk of keloid development. Based on MR analysis, CCND2 positively correlated with keloid risk [IVW OR: 1.410; 95% confidence interval (CI): 1.001–1.985, *P* = .049]. In contrast, KLF4 demonstrated a negative association, indicating a protective effect against keloid development (IVW OR: 0.492; 95% CI: 0.290–0.835, *P* = .009).

**Table 1.** MR results for the two core genes and keloid.

Outcome	Exposure	Method	nsnp	*b*	*P*-value	OR
Keloid	*CCND2*	MR Egger	4	0.942	.304	2.564
		Weighted median	4	0.353	.061	1.423
		IVW	4	0.343	.049	1.410
		Simple mode	4	0.258	.405	1.295
		Weighted mode	4	0.419	.136	1.520
Keloid	*KLF4*	MR Egger	4	−1.104	.503	0.332
		Weighted median	4	−0.554	.104	0.574
		IVW	4	−0.709	.009	0.492
		Simple mode	4	−0.596	.244	0.551
		Weighted mode	4	−0.531	.235	0.588

**Table 2.** Summary of MR analysis results for two core genes associated with keloid.

Exposure/method	nsnp	*P*-value	OR (95% CI)
*CCND2*			
Weighted median	4	.061	1.423 (0.984–2.058)
IVW	4	.049	1.410 (1.001–1.985)
*KLF4*			
Weighted median	4	.104	0.574 (0.295–1.120)
IVW	4	.009	0.492 (0.290–0.835)

**Figure 5.**
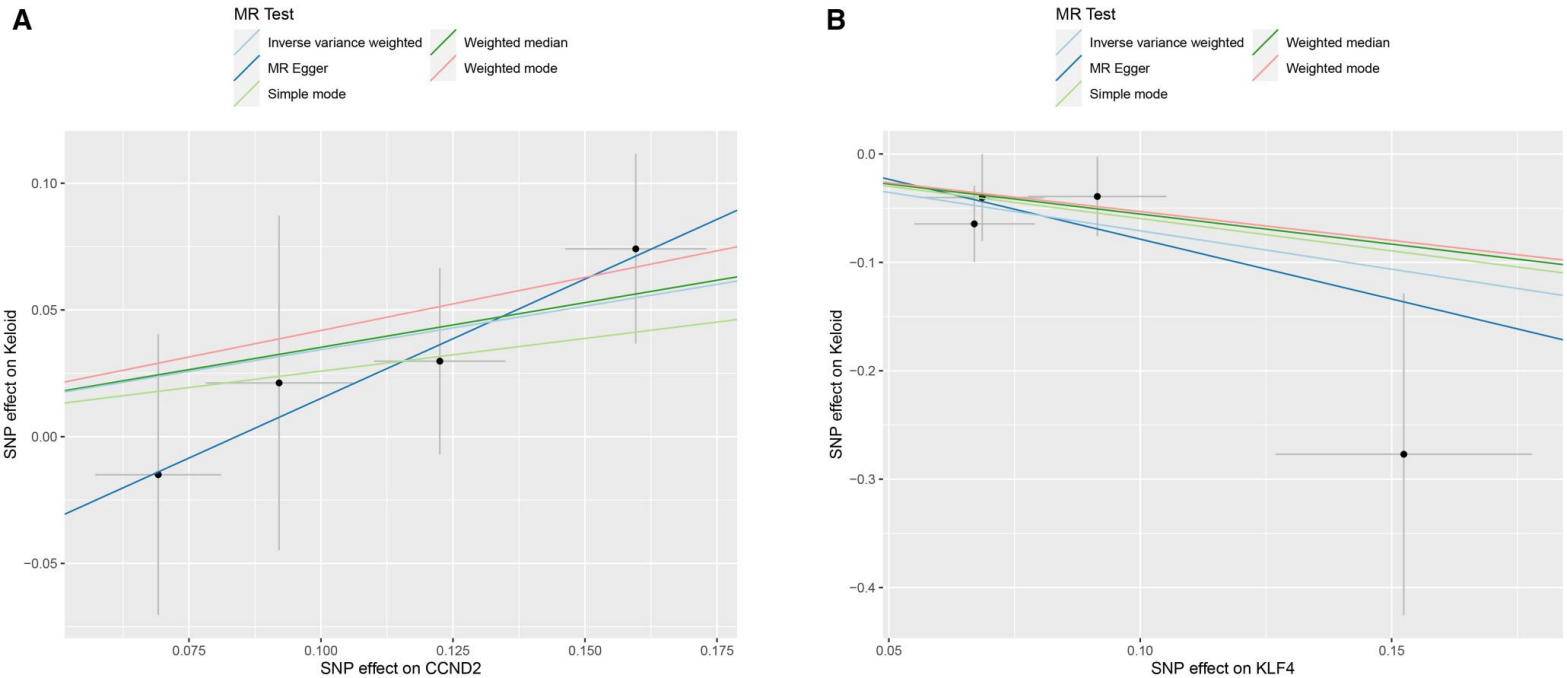
Scatter plots of the two core genes’ MR analyses for keloid. (A) Scatter plot for CCND2 and keloid MR analysis. (B) Scatter plot for KLF4 and keloid MR analysis. The horizontal axis represents the impact of SNPs on exposure factors (genes), the vertical axis represents the impact of SNPs on keloid, points represent SNPs, and colors represent the five methods used for Mendelian randomization, respectively.

**Figure 6.**
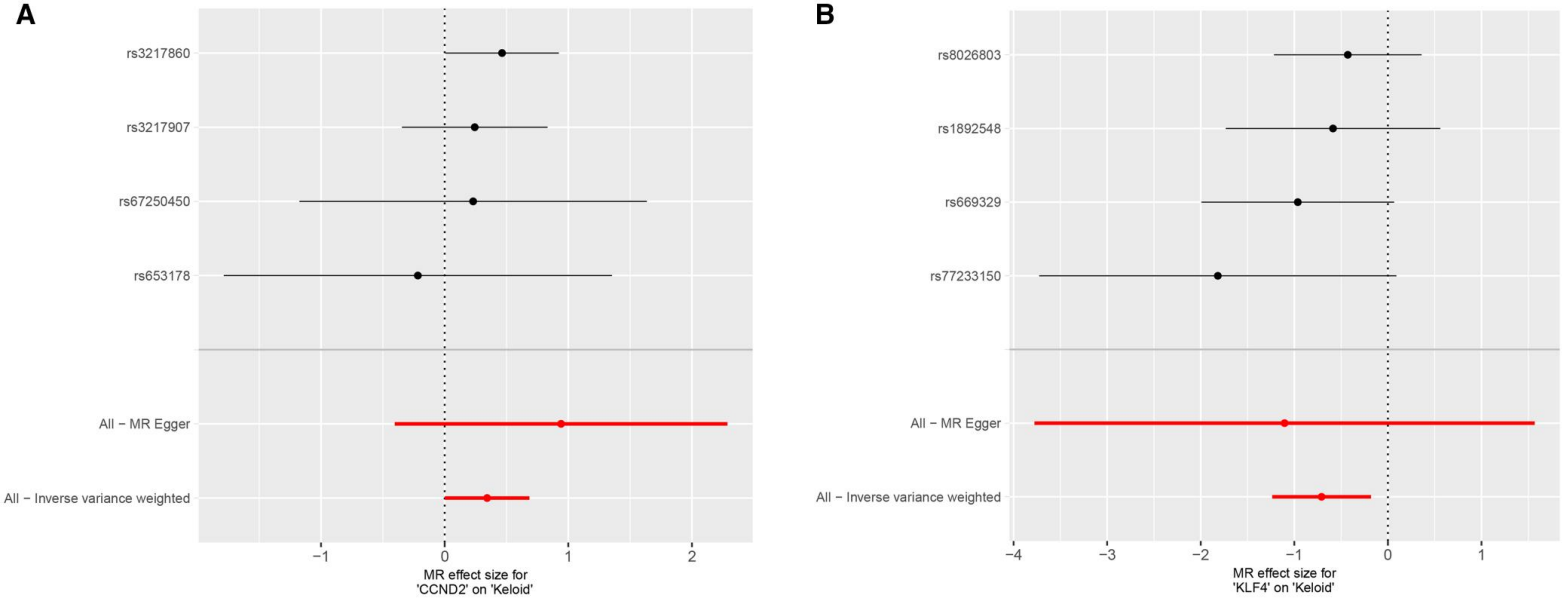
Forest plots for the two core genes. (A) Forest plot for CCND2 and keloid MR analysis. (B) Forest plot for KLF4 and keloid MR analysis.

To visualize these findings, scatter plots for the two core genes were generated (Fig. 5A and B), where lines of different colors represent the results of the five MR methods. The slope of each line reflects the extent of the gene’s influence on keloid development. In the scatter plot for CCND2, all five lines have slopes >0, confirming that CCND2 is a high-risk gene for keloid development. Conversely, the scatter plot for KLF4 shows that all five lines have slopes <0, indicating that KLF4 is a low-risk gene that reduces the risk of keloid development. To further illustrate these findings, forest plots for the two core genes were constructed (Fig. 6A and B). The forest plot for CCND2 shows that the combined effect sizes of SNPs associated with this gene are consistently >0, confirming its role as a high-risk gene for keloid development. In contrast, the forest plot for KLF4 demonstrates that the combined effect sizes of its associated SNPs are all <0, highlighting its function as a protective gene against keloid formation.

#### 3.5.2 Heterogeneity analysis, pleiotropy analysis, and sensitivity analysis

##### 3.5.2.1 Heterogeneity analysis

The MR results for the two core genes (CCND2 and KLF4) in relation to keloid were evaluated for heterogeneity using Cochran’s Q statistic, with additional tests performed using the MR Egger and IVW methods. The results are summarized in Table 3. The *P*-values for heterogeneity testing for CCND2 and KLF4 were >.05, indicating no significant heterogeneity in the data.

**Table 3.** Heterogeneity test results for MR analysis of two core genes with keloid.

Outcome	Exposure	Method	*Q*	*P*-value
Keloid	*CCND2*	MR Egger	0.080	.961
		Inverse variance weighted	0.891	.827
Keloid	*KLF4*	MR Egger	1.972	.373
		Inverse variance weighted	2.059	.560

##### 3.5.2.2 Pleiotropy analysis

The MR-Egger intercept method was applied to determine whether pleiotropic effects influenced the observed associations between the core genes and keloid. The results are shown in Table 4. For both CCND2 and KLF4, the *P*-values for pleiotropy testing were >.05, suggesting the absence of significant pleiotropic effects in the data.

**Table 4.** Results of Pleiotropy tests for the two core genes and keloid MR analysis.

Outcome	Exposure	egger_intercept	*P*-value
Keloid	*CCND2*	−0.079	.463
Keloid	*KLF4*	0.032	.795

##### 3.5.2.3 Sensitivity analysis

**Figure 7.**
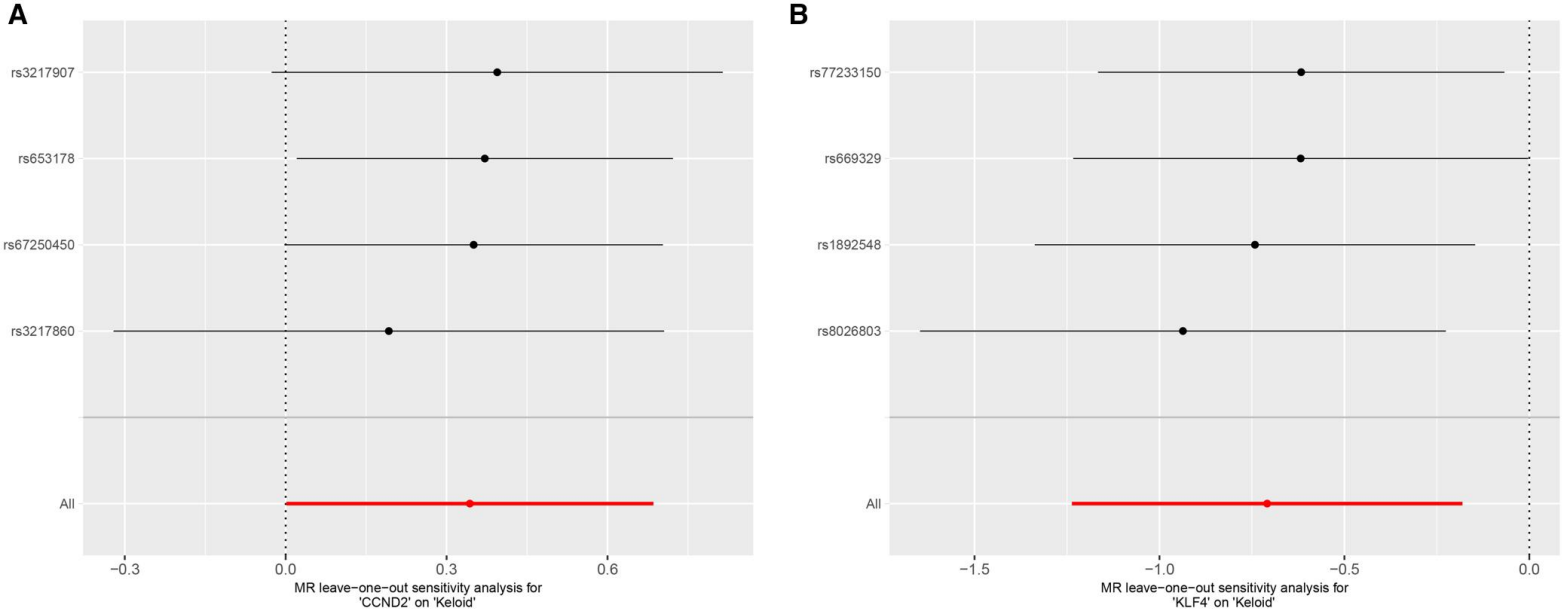
Leave-one-out sensitivity analysis for the two core genes. (A) Leave-one-out sensitivity analysis for CCND2 and keloid MR. (B) Leave-one-out sensitivity analysis for KLF4 and keloid MR.

To assess the robustness of the MR findings, leave-one-out sensitivity analyses were conducted for both core genes. The sensitivity plots for CCND2 and KLF4 are presented in Fig. 7A and B. In both cases, the removal of individual SNPs did not result in significant changes to the overall effect size compared to the combined effect size of all SNPs. These results confirm that the MR findings for CCND2 and KLF4 are robust and not overly influenced by any single SNP.

### 3.6 Functional and pathway enrichment analyses results of core genes

**Figure 8.**
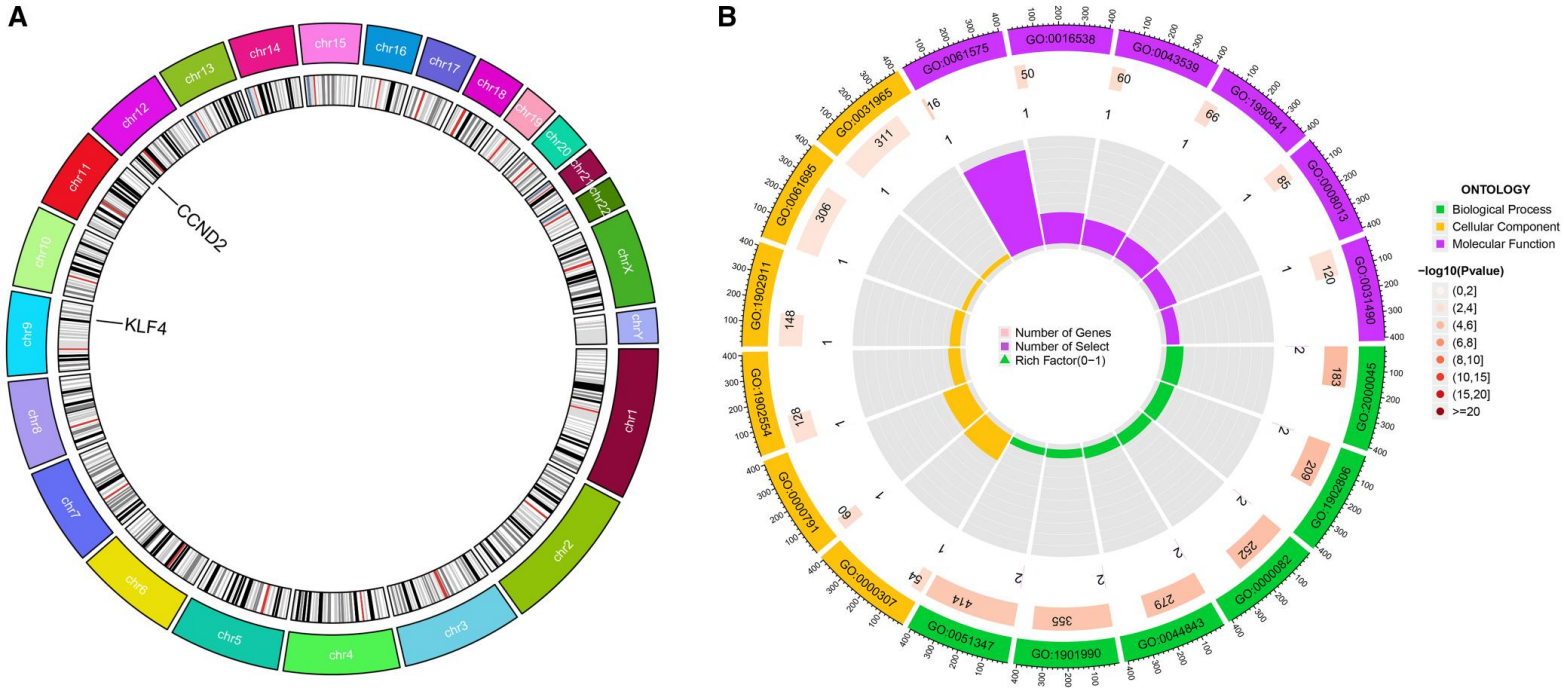
(A) Circular plot showing the chromosomal distribution of the two core genes. (B) Circular plot illustrating GO enrichment for core genes.

**Figure 9.**
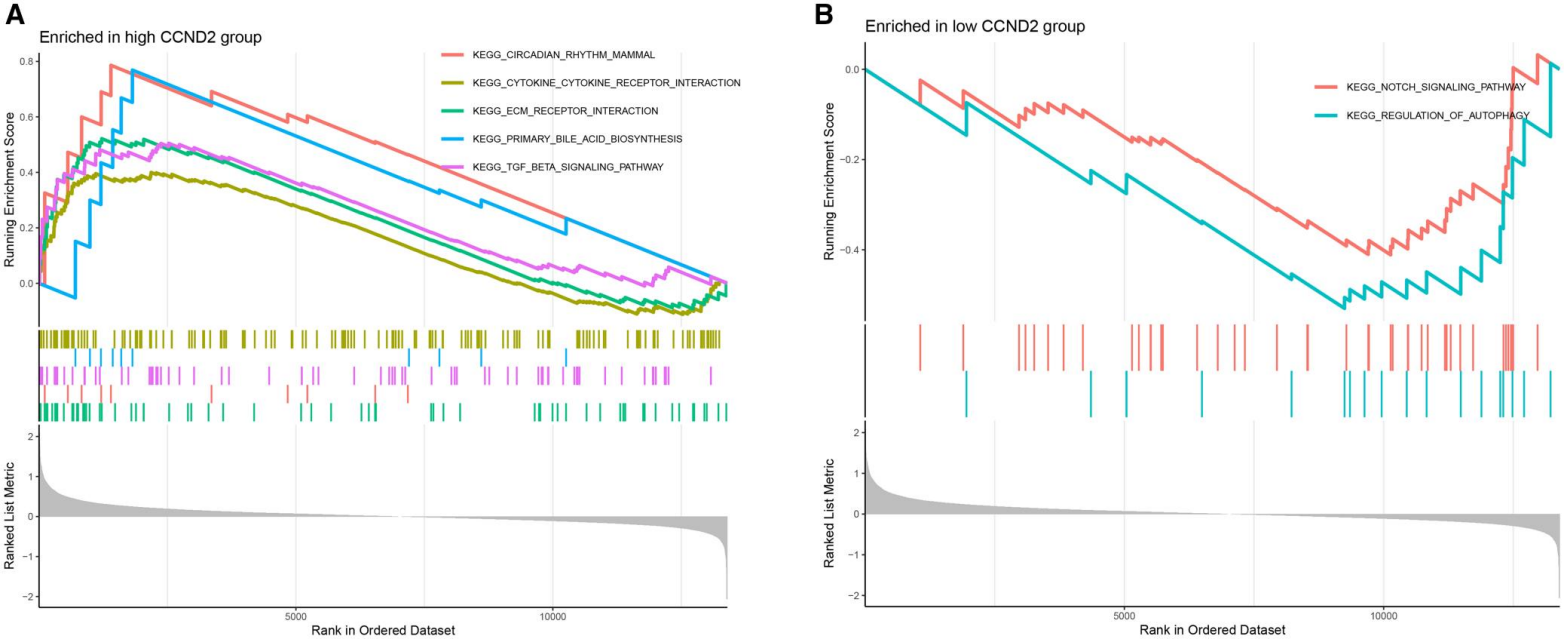
GSEA enrichment Plots for CCND2. (A and B) GSEA plots for CCND2.

**Figure 10.**
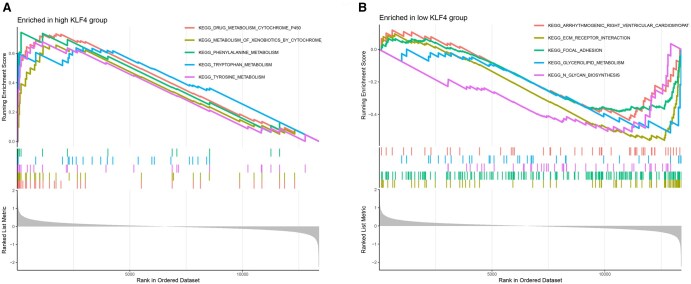
GSEA enrichment Plots for KLF4. (A and B) GSEA plots for KLF4.

The chromosomal distribution of the core genes was visualized, resulting in a circular plot (Fig. 8A) that illustrates the genomic locations of the core genes across chromosomes. A circle plot for core gene enrichment was also generated (Fig. 8B), showing significant enrichment of both core genes in BP. The specific pathways associated with their ID numbers are summarized in Table 5. Core genes were found to be significantly enriched in BP, such as cell cycle G1/S phase transition, regulation of mitotic cell cycle phase transition, and positive regulation of transferase activity. GSEA enrichment analysis was performed for the core genes, yielding enrichment plots (Figs 9A, B and 10A, B). For CCND2, the high-expression group was enriched in pathways such as cytokine-cytokine receptor interaction, ECM-receptor interaction, primary bile acid biosynthesis, and TGF-β signaling. In contrast, the low-expression group was enriched in the Notch signaling and autophagy regulation pathways. For KLF4, GSEA enrichment analysis revealed that the high-expression group was associated with pathways such as drug metabolism—cytochrome P450, phenylalanine metabolism, tryptophan metabolism, and tyrosine metabolism. The low-expression group, however, was enriched in pathways related to cardiac arrhythmias, cardiomyopathy, ECM-receptor interaction, adhesion, glycerolipid metabolism, and glycosaminoglycan biosynthesis.

**Table 5.** Specific descriptions corresponding to ID numbers in GO biological processes.

ID	Description	geneID	Count
GO:2000045	Regulation of G1/S transition of mitotic cell cycle	*CCND2/KLF4*	2
GO:1902806	Regulation of cell cycle G1/S phase transition	*CCND2/KLF4*	2
GO:0000082	G1/S transition of mitotic cell cycle	*CCND2/KLF4*	2
GO:0044843	Cell cycle G1/S phase transition	*CCND2/KLF4*	2
GO:1901990	Regulation of mitotic cell cycle phase transition	*CCND2/KLF4*	2
GO:0051347	Positive regulation of transferase activity	*CCND2/KLF4*	2
GO:1901987	Regulation of cell cycle phase transition	*CCND2/KLF4*	2

### 3.7 Immune infiltration analysis

**Figure 11.**
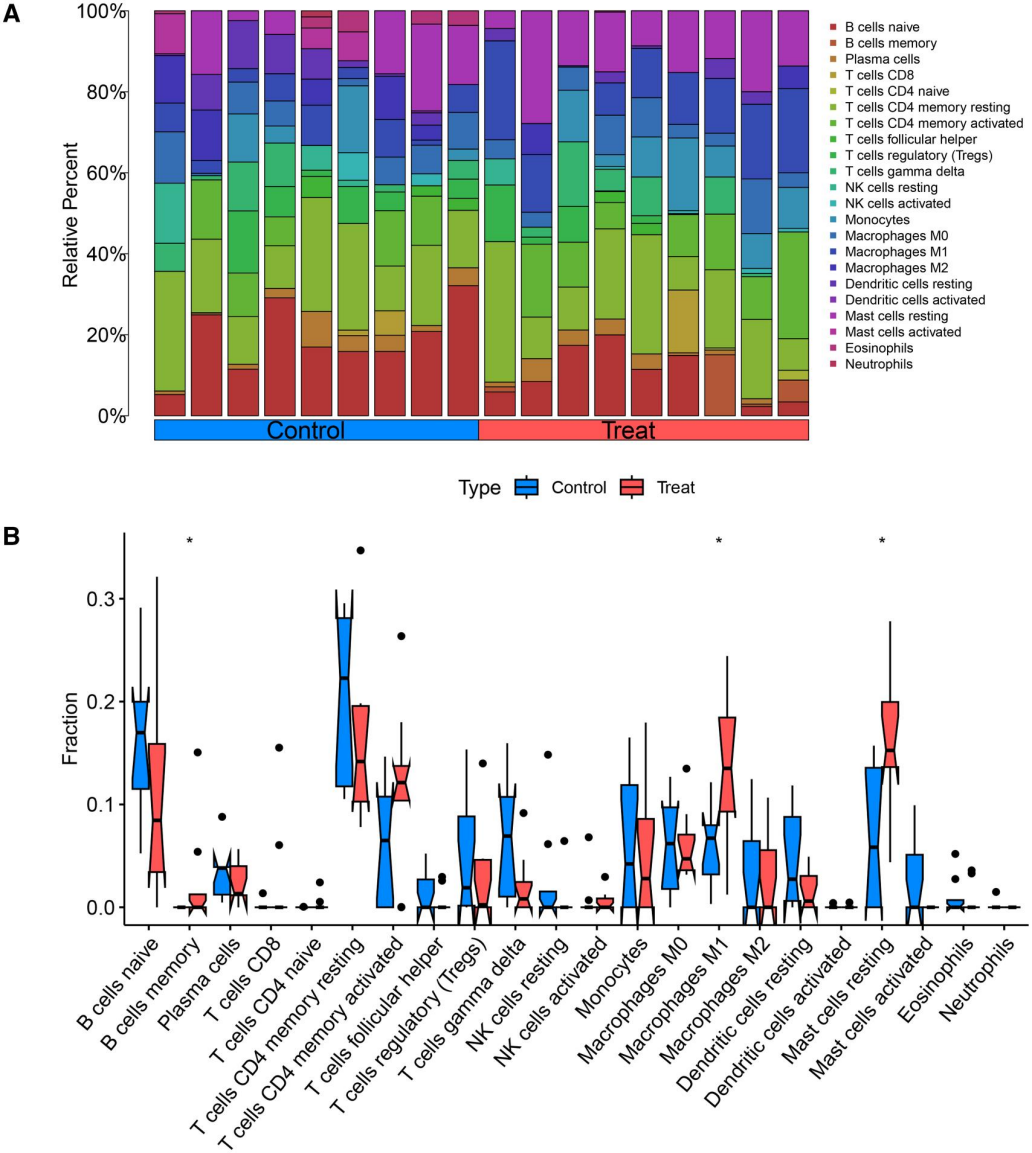
(A) Bar chart illustrating immune cell infiltration. (B) Boxplot showing differential immune cell counts. Stars indicate that there is a difference in this immune cell between the experimental group and the control group. Three stars represent *P* < .001, two stars represent *P* < .01, and one star represents *P* < .05.

**Figure 12.**
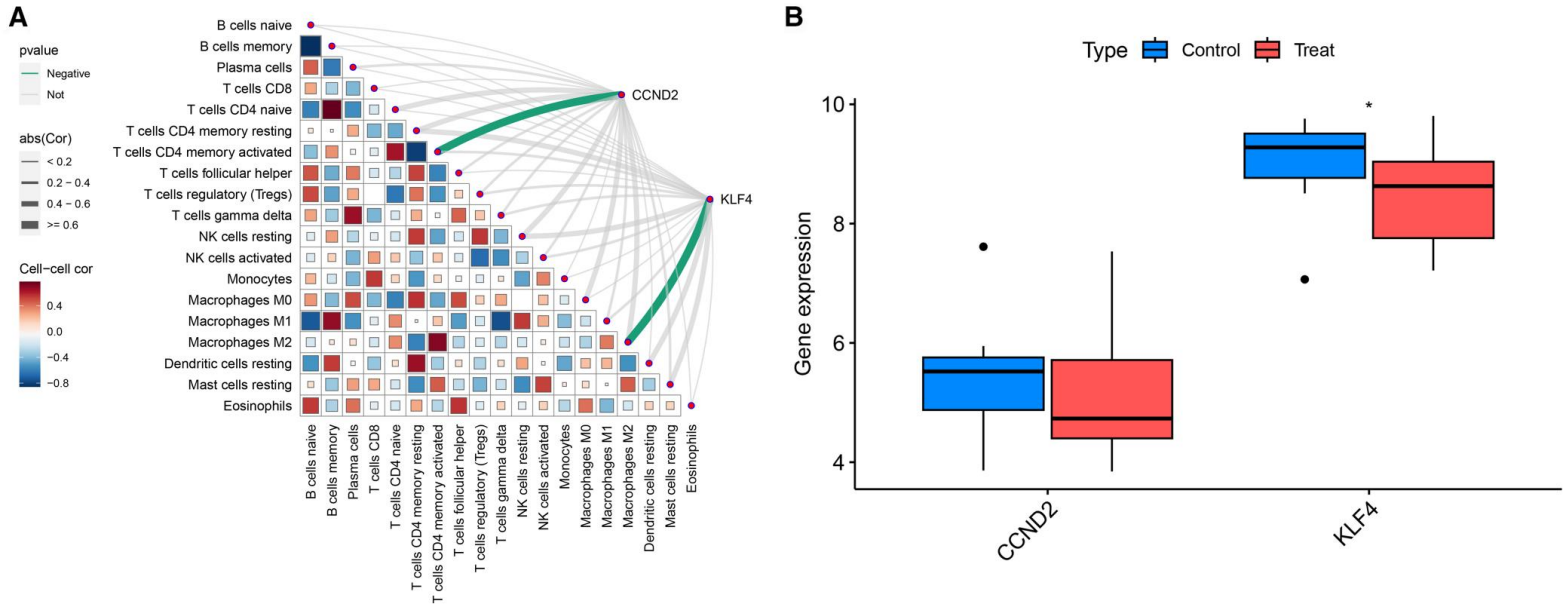
(A) Correlation plot between immune cells and core genes. (B) Differential expression analysis of core genes in the validation cohort.

Immune cell infiltration analysis was performed on the combined training dataset, generating a bar chart (Fig. 11A) that illustrates the proportion of immune cell types in each sample. An immune cell differential boxplot (Fig. 11B) revealed significant differences (*P* < .05) in the levels of B cells memory, Macrophages M1, and Mast cells resting between the experimental and control groups. These immune cell types were more highly expressed in the keloid experimental group. A correlation analysis was conducted to investigate the correlation between the core genes and immune cells, and the results were visualized in a correlation plot (Fig. 12A). The analysis revealed a negative correlation between CCND2 and T cells CD4 memory activated and a negative correlation between KLF4 and Macrophages M2.

### 3.8 Differential analysis results of core genes in the validation cohort

The differential expression of the two core genes was examined using the combined GSE7890 and GSE145725 datasets as the validation cohort. A boxplot (Fig. 12B) was generated, showing a significant difference in the expression of KLF4 within the validation cohort.

### 3.9 Expression of core genes in pan-cancer of TCGA and their impact on prognosis

**Figure 13.**
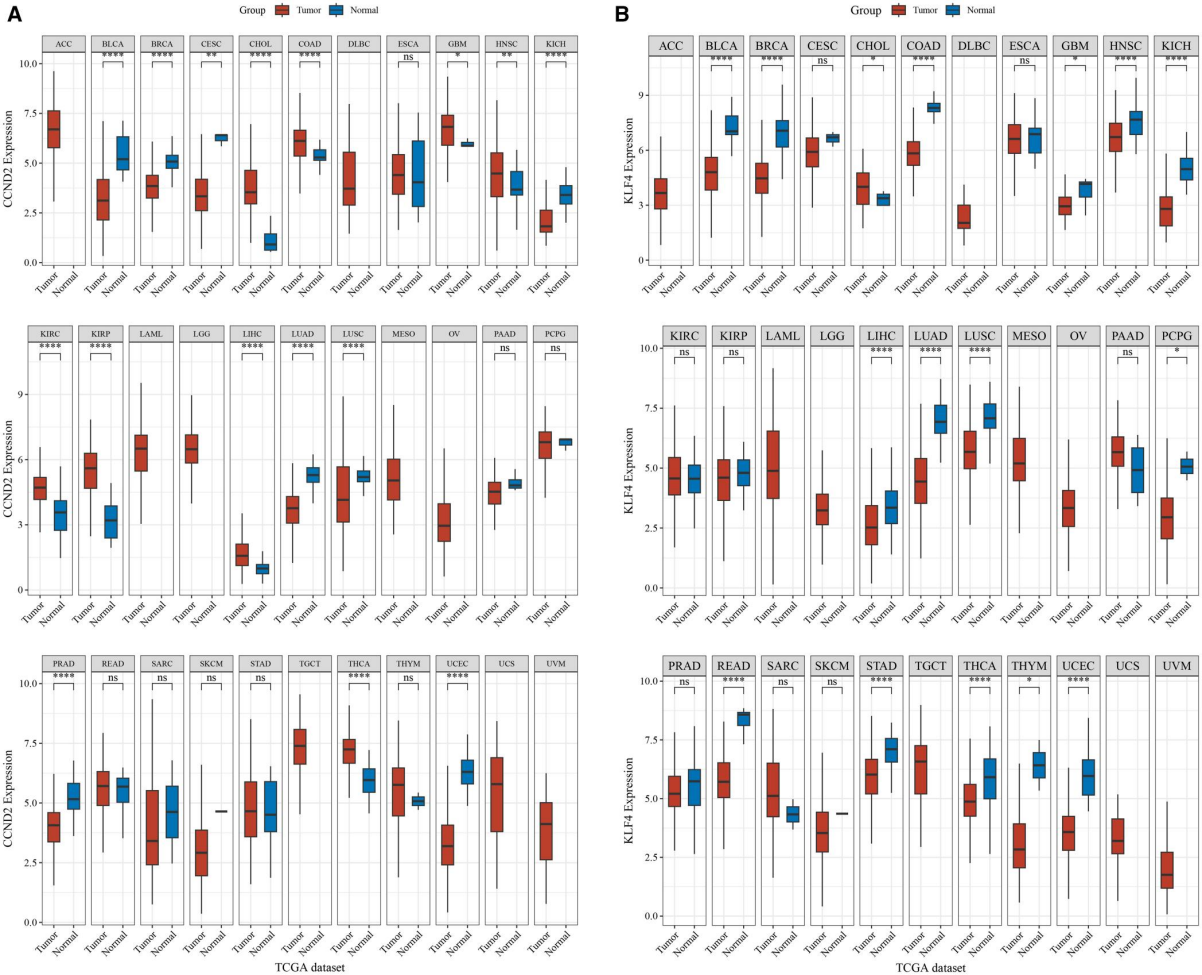
Boxplots of core gene expression distributions in tumor and normal tissues. (A) Expression of CCND2 across pan-cancers in the TCGA database. (B) Expression of KLF4 across pan-cancers in the TCGA database.

**Figure 14.**
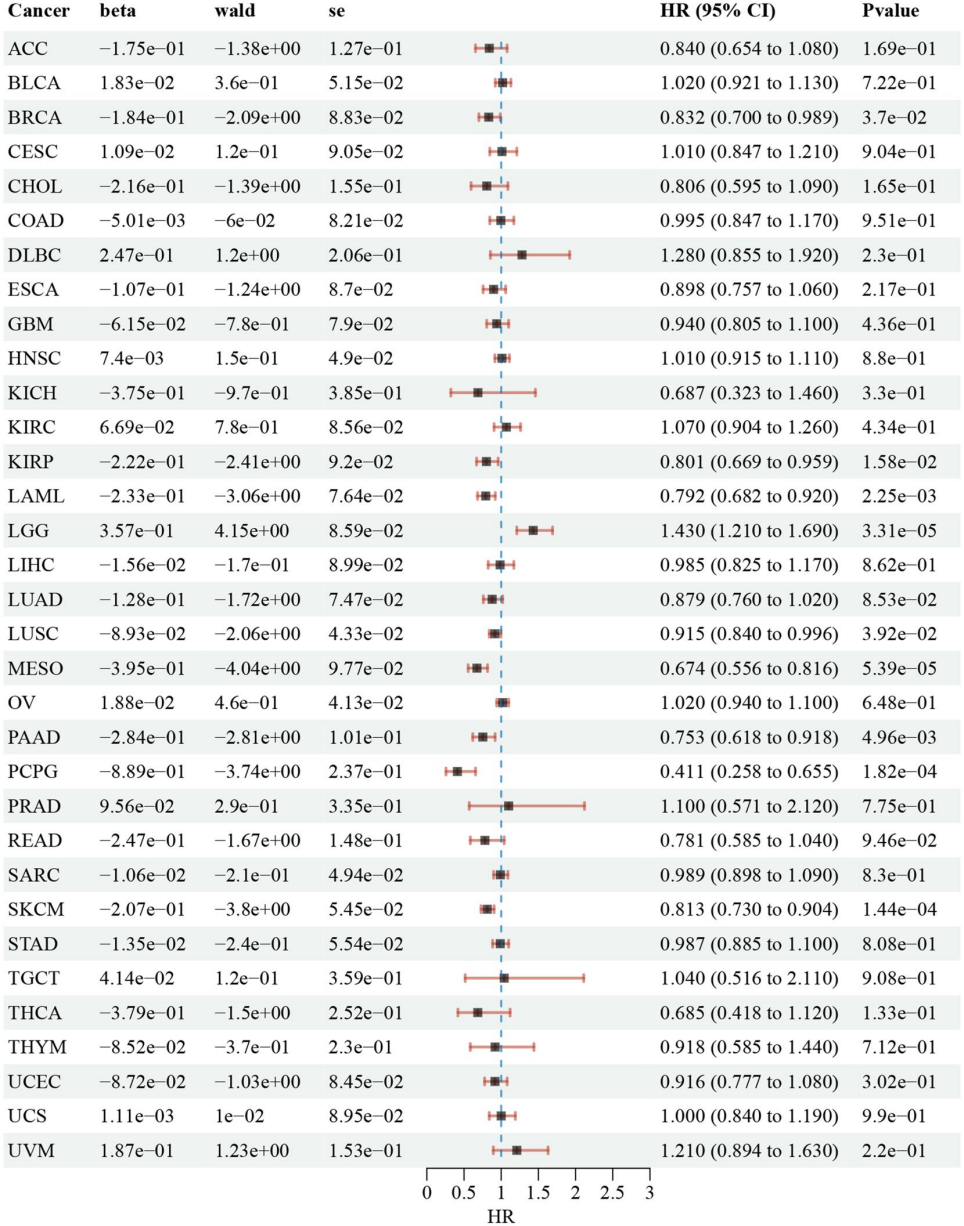
Cox regression analysis showing the relationship between CCND2 expression and patient prognosis in 33 cancer types.

**Figure 15.**
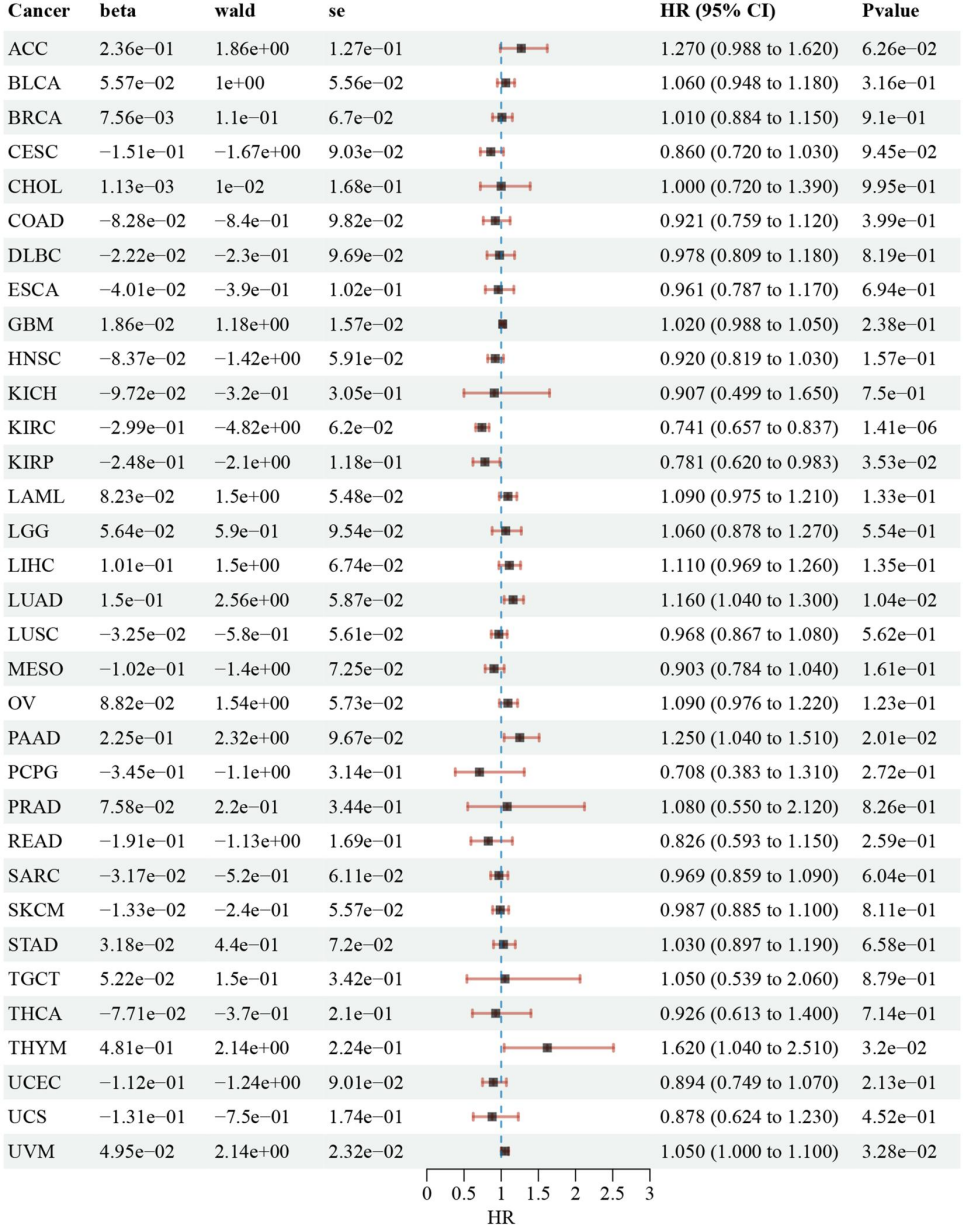
Cox regression analysis showing the relationship between KLF4 expression and patient prognosis in 33 cancer types.

The association between the expression of the two core genes and the occurrence and progression of 33 tumor types was further investigated using data from TCGA. CCND2 was found to be highly expressed in several cancers, including cholangiocarcinoma (CHOL), colon adenocarcinoma (COAD), glioblastoma multiforme (GBM), head and neck squamous cell carcinoma (HNSC), kidney renal clear cell carcinoma(KIRC), kidney renal papillary cell carcinoma (KIRP), liver hepatocellular carcinoma (LIHC), lung squamous cell carcinoma (LUSC), and thyroid carcinoma (THCA), as shown in Fig. 13A. Additionally, KLF4 was highly expressed in CHOL, as depicted in Fig. 13B. This study also explored the prognostic impact of CCND2 and KLF4 expression on various cancer types. Cox regression analysis identified CCND2 as a risk factor for HNSC and KIRC (Fig. 14). Similarly, KLF4 was identified as a risk factor for CHOL (Fig. 15).

## 4 Discussion

Keloids are fibroproliferative skin disorders characterized by excessive fibroblast proliferation and abnormal ECM accumulation. Clinically, keloid lesions can invade surrounding normal tissues, often adopting dumbbell-shaped or crab-foot-like appearances. These lesions are associated with persistent growth, redness, itching, pain, and a high recurrence rate. The pathogenesis of keloids is highly complex, with genetic factors and mutations increasingly recognized as critical contributors to keloid formation (Huang *et al.* 2022). Keloids exhibit diverse genetic patterns, with autosomal dominant inheritance being the most prominent (Shih and Bayat 2010). This disorder also shows familial clustering and predisposition, particularly among individuals with darker skin tones who are at a higher risk of developing keloids. To deepen the understanding of the genetic mechanisms underlying keloid formation, this study used bioinformatics and MR approaches. These methodologies identified core genes associated with keloids, providing novel insights into the pathogenesis of the disease. By uncovering the roles of these core genes, the findings may facilitate the development of targeted therapies and prognostic strategies. Through the application of advanced bioinformatics and MR methods, this study seeks to contribute to the growing body of keloid research and ultimately improve patient outcomes.

This study initially combined the GSE92566 and GSE121618 datasets to identify keloid-related DEGs. This analysis resulted in the identification of 244 upregulated and 137 downregulated DEGs. Subsequently, we conducted an MR study using the gene eQTL database as the exposure factor and keloid GWAS data (ebi-a-GCST90018874) as the outcome factor. This analysis identified 93 high-risk candidate genes and 109 low-risk candidate genes. By intersecting the DEGs with the MR-identified candidate genes, we identified two genes of particular interest: CCND2, an upregulated gene, and KLF4, a downregulated gene.

Further MR analysis of these two intersection genes with keloid GWAS data revealed significant associations. Specifically, CCND2 was associated with keloids with an IVW OR of 1.410 (95% CI: 1.001–1.985, *P* = .049), suggesting a potential causal relationship. KLF4 was significantly associated with keloids with an IVW OR of 0.492 (95% CI: 0.290–0.835, *P* = .009), indicating a causal link. Both genes had *P*-values below .05, confirming their causal associations with keloids. Consequently, CCND2 and KLF4 were identified as core genes in our study, providing valuable insights into the pathogenesis of keloids and highlighting potential targets for therapeutic intervention. The beta value for CCND2 was >0, and the OR was >1, establishing it as a risk factor. In contrast, the beta value for KLF4 was <0, and the OR was <1, classifying it as a protective factor. We performed heterogeneity and pleiotropy analyses on the MR results to validate these findings. The *P*-values for these tests were >.05, indicating no significant heterogeneity or pleiotropy and confirming the reliability of the MR analysis. Furthermore, a leave-one-out sensitivity analysis demonstrated that the removal of any single SNP from the analysis did not significantly alter the results, further supporting the robustness of our findings. The analysis of CCND2 and KLF4 is detailed below.

Cyclin D2 (CCND2), a member of the cyclin family, is a critical cell cycle regulator. Our functional enrichment analysis corroborates its pivotal role in driving the transition from the G1 phase to the S phase of the cell cycle. Overexpression of CCND2 has been linked to tumorigenesis and cancer progression, with aberrant expression or mutations identified in cancers such as thyroid cancer, lung cancer, and breast cancer (Park *et al.* 2010, Chen *et al.* 2018, Hung *et al.* 2018, Xia *et al.* 2018, Yuan *et al.* 2023, Jia *et al.* 2024). These findings suggest that CCND2 may be a potential biomarker for various malignancies. Beyond its implications in cancer, studies in animal models have demonstrated that CCND2 can activate the cell cycle in myocardial cells following myocardial infarction in mice and pigs, indicating its role in myocardial cell development (Sun *et al.* 2023). Additionally, CCND2 has been implicated in human brain growth, with mutations in this gene associated with conditions such as microcephaly and dwarfism (Pirozzi *et al.* 2021). Our GSEA enrichment analysis further highlights the involvement of CCND2 in key pathways, including cytokine receptor interaction, ECM receptor interaction, primary bile acid biosynthesis, and TGF-β signaling. These findings suggest that CCND2 contributes to the pathogenesis of keloids by regulating cell cycle progression, cell proliferation, and cytokine interactions. Specifically, CCND2’s role in driving cell cycle progression and proliferation and its influence on cytokine signaling may underlie the excessive fibroproliferation and ECM deposition characteristic of keloid lesions. In summary, CCND2 emerges as a pivotal factor in keloid development, acting through its multifaceted effects on cell cycle regulation, proliferation, and cytokine signaling. Further research into the precise mechanisms by which CCND2 influences keloid formation could provide valuable insights for the development of targeted therapies.

Krüppel-like factor 4 (KLF4) is a zinc finger-containing transcription factor crucial in regulating cell growth, proliferation, and differentiation. It exerts its transcriptional regulatory effects through various mechanisms, including phosphorylation, acetylation, methylation, and ubiquitination (Ghaleb and Yang 2017). KLF4 has been shown to induce cell cycle arrest and enhance cell survival by inhibiting apoptosis (Kuruvilla *et al.* 2016). In the context of cancer, KLF4 regulates several malignancies, including lung cancer, colon cancer, hepatocellular carcinoma, and meningiomas (Tsytsykova *et al.* 2022, Chen *et al.* 2023, He *et al.* 2023, Zheng *et al.* 2023). Within the skin, KLF4 is highly expressed in the epidermis and is integral to maintaining skin barrier function, with ectopic expression accelerating the formation of the epidermal permeability barrier (Jaubert *et al.* 2003). KLF4 deficiency has been associated with increased susceptibility to skin tumorigenesis (Li *et al.* 2012a). In wound healing, KLF4 promotes the generation of fibroblasts from bone marrow-derived suppressor cells, facilitating skin repair (Ou *et al.* 2015). Notably, KLF4 expression is reduced in fibroblasts, and transfection studies have demonstrated its ability to attenuate the trans-differentiation of fibroblasts into myofibroblasts, thereby inhibiting fibrosis development (Wang *et al.* 2022). This finding underscores the critical role of KLF4 in regulating the formation of proliferative scars, including keloids, aligning with observations from our study. GSEA enrichment analysis revealed that KLF4 is underexpressed in ECM receptor interactions and adhesion pathways. This finding suggests that KLF4 may mitigate keloid development by modulating cell growth, inhibiting fibrosis progression, and regulating ECM receptor interactions. Specifically, KLF4’s ability to suppress fibrosis and the transdifferentiation of fibroblasts into myofibroblasts could limit the excessive ECM deposition that defines keloid lesions. In conclusion, KLF4 emerges as a critical regulator of keloid formation through its multifaceted effects on cell growth, fibrosis inhibition, and ECM receptor interactions. Further research into the precise mechanisms by which KLF4 influences keloid development may pave the way for novel therapeutic strategies to address this debilitating condition.

We also conducted an immune infiltration analysis on the training cohort sample data to explore the involvement of immune cell subtypes in keloid pathogenesis. This analysis revealed significant differences in the infiltration of B cells memory, Macrophages M1, and Mast cells resting between the experimental and control groups. Notably, increased infiltration of these immune cells was observed in keloid samples, suggesting their critical roles in keloid development. Previous studies have highlighted the importance of macrophages in keloid formation, demonstrating that these cells regulate the microenvironment from the early stages of wound healing to the later stages of scar formation (Xu *et al.* 2020). Macrophages in keloid tissues are highly activated and promote the differentiation of Treg cells through the upregulation of Foxp3 expression (Jin *et al.* 2018), which aligns with our immune infiltration results. Furthermore, we analyzed the correlations between the two core genes and immune cell subtypes. CCND2 was found to be negatively correlated with T cells CD4 memory activated, while KLF4 exhibited a negative correlation with Macrophages M2.

Lastly, we validated the two core genes using a combined dataset from GSE7890 and GSE145725 as the validation cohort. Our analysis confirmed that KLF4 exhibited significant differences in the validation cohort, further supporting its relevance in keloid pathogenesis.

In our exploration of the core genes CCND2 and KLF4, we identified associations between their expression and the development of specific tumor types. To investigate this further, we performed a pan-cancer analysis using TCGA data to examine the relationship between these keloid-related core genes and tumorigenesis. The analysis revealed that high expression of CCND2 promotes proliferation and invasion in head and neck squamous cell carcinoma and clear cell renal cell carcinoma, establishing CCND2 as a risk factor for these malignancies. Similarly, elevated expression of KLF4 enhances proliferation and invasion in cholangiocarcinoma, suggesting that KLF4 acts as a risk factor in this cancer type.

In the future, further clinical studies and experimental investigations are essential to validate the roles of these two core genes in keloid formation and progression. We hope our research provides new insights into the pathogenesis and treatment of keloids, offering potential therapeutic targets and strategies for this challenging condition.

## 5 Conclusion

This study explored the molecular mechanisms underlying keloids using an integrated approach that combined bioinformatics analysis, MR methods, and data validation strategies. The findings reveal a significant positive correlation between high expression of CCND2 and keloid formation, identifying CCND2 as a risk factor for keloids. Conversely, high expression of KLF4 is negatively correlated with keloid risk, suggesting that KLF4 serves as a protective factor. Validation experiments using the combined dataset from GSE7890 and GSE145725 confirmed that KLF4 exhibited significant differences in the validation cohort, further reinforcing its potential role in keloid pathogenesis. This study not only highlights the pivotal roles of CCND2 and KLF4 in the development of keloids but also provides novel molecular targets and a theoretical foundation for the prevention and treatment of keloids.

## Data Availability

The public data that support the findings of this study are available from https://www.ncbi.nlm.nih.gov/geo/ and https://gwas.mrcieu.ac.uk/.
